# Knowledge, Attitude, and Practices on Food Safety among Food Handlers Working in Public Food Service Establishments in Lemi Kura Subcity, Addis Ababa, Ethiopia

**DOI:** 10.1155/2024/2675894

**Published:** 2024-01-23

**Authors:** Yordanos Fekadu, Mebrie Zemene Kinde, Gashaw Getaneh Dagnaw, Bereket Dessalegn, Haileyesus Dejene, Abebe Tesfaye Gessese

**Affiliations:** College of Veterinary Medicine and Animal Sciences, University of Gondar, P.O. Box 196, Gondar, Ethiopia

## Abstract

Foodborne diseases, resulting from poor food handling and sanitation practices, are common public health problems globally. The primary contributing factors to potential foodborne disease outbreaks are often attributed to the poor perception and practices of food handlers regarding food safety. This study is aimed at assessing the knowledge, attitude, and practices of food handlers working in public food service establishments in Lemi Kura subcity, Addis Ababa, Ethiopia. A cross-sectional study was conducted from December 2022 to September 2023, involving 400 food handlers from 20 randomly selected public food service establishments. Data were collected using a structured interview-administered questionnaire and an observational checklist. The collected data were entered into Microsoft Excel and then transferred to SPSS version 20 for analysis. Descriptive statistics were employed to summarize the data, and Pearson's chi-square test was used to evaluate the association of sociodemographic factors with the knowledge, attitude, and practices of food handlers towards food safety. Of the 400 food handlers, 65.5% had good knowledge about food safety. All food handlers were knowledgeable that washing hand before handling food will reduce risk of poisoning, bacteria are the main cause of food poisoning, and temperature plays a big role in bacterial growth. This study also revealed that 65.3% of the food handlers had good attitude towards food safety and 55.3% of food handlers had good food handling practice. Based on the observation, 38.5% of food handlers had good food handling practice. Taking training, age, and work experience of food handlers have statistically significant (*P* < 0.05) association with good attitude towards food safety. Additionally, taking training, educational level, employment, and work experience of food handlers have statistically significant (*P* < 0.05) association with good practice towards food safety. However, there was no statistically significant association between all sociodemographic factors and knowledge about food safety. Good knowledge and attitude were also associated with good food handling practices of the respondents. Based on the observation, there was a statistically significant association between employment status and good food handling practice. In conclusion, the findings suggest the necessity for implementing formal educational and training programs aimed at positively influencing the knowledge and attitude of food handlers, subsequently improving their food handling practices.

## 1. Introduction

Food is vital for human health and overall well-being. Various factors, including contamination, impact the health of individuals worldwide. While developing nations often face more significant challenges, developed countries also experience issues related to food safety. Despite technological advancements, the occurrence of food-related diseases persists [1].

The World Health Organization reported that annually, up to 600 million individuals worldwide become sick, with 420,000 fatalities attributed to the consumption of contaminated food. In the United States, approximately 48 million people experience foodborne illnesses each year, leading to 128,000 hospitalizations and 3,000 deaths. Regions such as Africa and Southeast Asia are identified with the highest rates of incidence and death related to foodborne diseases. The recurrence of foodborne disease has increased worldwide concern about food hygiene and safety among food handlers [2].

Due to the presence of contaminants in food, there exists a close relationship between food safety and food security. Developing countries experience elevated levels of food contamination compared to the United States and Europe [3]. In these regions, inadequate food storage practices contribute to contamination and limited access to safely processed foods, thereby contributing to malnutrition and hunger [4–6]. Within public food establishments, food handlers play a crucial role in introducing contaminants, serving as carriers for various pathogenic organisms, whether biologically or physically [7].

The occurrence of over 200 different foodborne diseases and illnesses is attributed to the combined influences of food production, processing, distribution, transportation, and preparation [8]. The challenges posed by food globalization further exacerbate this issue. The ongoing global challenge of foodborne illnesses is dynamic, influenced by factors such as international food trade, advancements in food production technologies, the emergence of new pathogens, and evolving consumer behaviors and preferences [9, 10].

To mitigate foodborne illnesses, it is crucial to recognize that many cases stem from improper food handling techniques, often occurring in both home kitchens and food establishments such as restaurants [11, 12]. A fundamental element in enhancing food safety is education. Without a thorough understanding of proper food safety practices and handling procedures, reducing the incidence of foodborne illnesses becomes challenging [13]. To address this concern, it is imperative to develop educational interventions specifically targeting food safety behaviors and risks [14].

Hence, the majority of studies suggest that although having knowledge is crucial for food hygiene, possessing knowledge alone does not guarantee the adoption of safe food handling practices [15]. Various factors contributing to consumers' reluctance to adopt safe food handling behaviors have been recognized, such as a diminished perception of risk, a low sense of susceptibility, optimistic bias, reliance on heuristics, and entrenched habitual practices. The utilization of behavior change theories could be beneficial in comprehending how these factors influence the adoption of safe food handling behaviors, especially among young individuals [16].

In Ethiopia, information about the level of foodborne disease due to improper food safety in food and drink service establishments is not satisfactory. But from the different settings of the country, some studies show that there is a high sanitary problem in catering establishments [17–19]. A comprehensive analysis of studies conducted in Ethiopia from January 2000 to July 2020 revealed an overall random pooled prevalence estimate of 8% for bacterial foodborne pathogens, as reported by [20]. In Northern Ethiopia, the overall prevalence of foodborne protozoa infection was 45.3% [21]. The overall health burden due to foodborne zoonotic diseases in three towns (Gondar, Lalibela, and Debark) of Amhara regional state was estimated to be 89.9 DALYs per 100,000 populations per year [22]. These reports highlight the significance of addressing and managing foodborne diseases in Ethiopia.

There are some studies on food handlers' knowledge, attitude, and practices towards food safety in different parts of Ethiopia. Food handlers had a good level of knowledge (73.8%), positive attitude (64.4%), and good hygienic practices (42.3%) in Southern Tigray [23]. [24] also reported that 34.1% has good knowledge and 54% has good food safety practice among food handlers in Debre Markos town, Northwest Ethiopia. [25] reported 72.4% good knowledge level, 94.6% positive attitude, and 83.7% poor food safety practice among food handlers in Bishoftu City. According to [26], a pooled proportion of good food hygiene practices among food handlers in Ethiopia was 50.5% from studies conducted until February 24, 2022.

There are a few reports regarding the knowledge, attitude, and practice (KAP) of food handlers about food safety in some subcities of Addis Ababa; 93.7% of food handlers had adequate knowledge of food borne diseases, 52.3% of food handlers had a poor food handling practice in Addis Ababa University students' cafeteria [27], and 27.4% of food handlers had good food hygiene practices in Bole subcity [28]. Knowledge of food safety was significantly related to age, education level, and work experience of food handlers. Food safety practice was significantly associated with age in the study conducted in Yeka subcity [29], and 40.2% of food handlers had good food handling practice in Yeka subcity [30], which indicated a low awareness and practice towards food safety. However, there was no similar study in Lemi Kura subcity of Addis Ababa as it is a newly formed subcity. Therefore, the objective of this research was to assess the knowledge, attitude, and practices of food handlers towards food safety in Lemi Kura subcity, Addis Ababa, Ethiopia.

## 2. Materials and Methods

### 2.1. Study Area

The study was conducted in Lemi Kura subcity, Addis Ababa, Ethiopia, from December 2022 to September 2023. Lemi Kura subcity is one of the eleven subcities found in Addis Ababa, which is a newly formed subcity. The subcity's overall population stood at 312,209 and was comprised of 10 woredas [31]. As per the information provided by the Food and Drug Administration (FDA) office in Lemi Kura subcity, there are 353 officially registered food establishments, including cafeterias, restaurants, and nonstarred hotels.

### 2.2. Study Design

A cross-sectional study was conducted from December 2022 to September 2023 to assess the knowledge, attitude, and practices of food handlers towards food safety.

### 2.3. Study Population

All food handlers working at public food establishments in Lemi Kura subcity were considered the study population. Food handlers working in preparation, management, butchery, and service areas of food establishments including restaurants, hotels, cafeterias, milk distributers, and butcher houses were included in the study. Food handlers who have worked less than six months in food establishments and who are on annual leave and seriously ill during data collection period were excluded in the study. The respondents were grouped into three age categories (18-21 years, 22-25 years, and >25 years). In addition, based on their experience, respondents were categorized in to three groups (<1 year, 1-3 years, and >3 years) [32].

### 2.4. Sample Size Determination and Sampling Method

The sample size for the study was determined based on the description of [33] and by taking the expected prevalence of 50% as there was no previous study in Lemi Kura subcity. Reports from other subcities of Addis Ababa were not included in the sample size calculation for this study. This decision was made because the educational levels, access to information, and healthcare infrastructure in Lemi Kura subcity differ significantly from those in the other subcities. Notably, Lemi Kura subcity incorporates new kebeles from rural areas. The confidence interval of 95% and required absolute precision of 5% were considered. Then, the minimum required sample size was calculated using the following formula:

(1) $$\begin{array}{c} N=\frac{{\left(1.96\right)}^{2}\text{Pexp }\left(1-\text{Pexp}\right)}{d^{2}}, \end{array}$$

where *N* is the sample size, Pexp is the expected prevalence, and *d* is the required precision. By substituting the values in the formula and taking *d* = 0.05,

(2) $$\begin{array}{c} N=\frac{\left(1.96\right)20.5\left(1-0.5\right)}{{\left(0.05\right)}^{2}}=384. \end{array}$$

Even though the calculated sample size was 384, this study included a total of 400 respondents by considering 5% nonresponse rate.

From the 11 subcities of Addis Ababa, Lemi Kura subcity was selected purposively for the reason that it is a newly formed subcity and there was no previous research conducted. From the 10 woredas of the subcity, four woredas were selected using simple random sampling method. From the four woredas, 20 food establishments were selected using simple random method. All individuals responsible for food handling within the chosen food establishments were incorporated into the study after providing their consent following a clear explanation of the research's objectives. Those food handlers who chose not to take part in the study were not considered.

### 2.5. Data Collection Tool and Procedure

A structured questionnaire was employed to conduct face-to-face interviews, gathering data on the food handlers' knowledge, attitudes, and practices related to food safety. The questionnaire was structured into four parts: sociodemographic part with seven questions, food safety knowledge part with eight questions, food safety attitude part with nine questions, and food safety practice part with 25 questions. The questionnaire was developed from previous studies conducted [19, 34, 35]. Additionally, observation checklist was employed for collecting data on food handling practice of food handlers. In order to assure the quality of the data, a pretest of data collection instrument was carried out on 5% of the total sample size outside the study area.

Sociodemographic section of the questionnaire consisted of age, educational level, position, experience, employment status, and training related to food handlers. The assessment of food safety knowledge involved nine closed-ended questions with two responses, “yes” or “no.” These questions primarily addressed topics such as the personal hygiene of food handlers, temperature control, bacterial growth, food poisoning, cross-contamination, food storage, and equipment hygiene. Food safety attitude was also assessed using nine closed-ended questions with 3 possible answers: “always,” “sometimes, “and “never”. Food safety practices were also assessed using 25 closed-ended questions with two possible answers: “yes” or “no”. Observational assessment was assessed using 17 closed-ended questions with two possible answers: “yes” or “no”.

### 2.6. Operational Definition

Food hygiene practices refer to the actions undertaken by food handlers to safeguard food from contamination, ensuring a secure food supply for consumers.

For food hygiene practice level, a score of one was assigned for each “yes” response indicating standard practice and zero for each “no” response. Food handlers surpassing the mean total score were classified as having “good food safety practices,” while those falling below the mean were categorized as having “poor food safety practices” [32, 36].

One score was given for every standard observation and zero for every unsafe observation. Food handlers with a total score greater than the mean were considered to have “good food safety practices,” while those with a score less than the mean were considered to have “poor food safety practices.”

For food hygiene knowledge level, one point was assigned for each “yes” response, and zero points for “no” answers. The scores from these questions were then aggregated to generate a knowledge score. Food handlers achieving a total score surpassing the mean were characterized as having “good food safety knowledge,” while those with scores below the mean were labeled as having “poor food safety knowledge” [32, 37].

For food hygiene attitude level, a score of two was given for every “always” and one for every “sometimes” and zero for “never” responses. Food handlers with a total score greater than the mean were considered to have “good food safety attitude,” while those with a score less than the mean were considered to have “poor food safety attitude”.

### 2.7. Data Analysis

The data collected on the paper format was checked for any error, corrected, and then transferred to and stored in Microsoft Excel and then was transferred to SPSS version 20 for analysis. A descriptive analysis was employed to describe the percentages and number of distributions of the respondents based on sociodemographic characteristics and other relevant variables in the study. Pearson's chi-square test was used to evaluate the association of different sociodemographic factors with knowledge, attitude, and practice of food handlers towards food safety. Throughout all the statistical analyses conducted, a confidence level of 95% was applied, and a *P* value less than 0.05 (at a 5% level of significance) was regarded as statistically significant.

## 3. Results

### 3.1. Sociodemographic Characteristics of Food Handlers

In this study, a total of 400 food handlers were participated. Slightly more than half of the food handlers (51.5%) were male. The mean age of participants was 29.68years (SD = 6.71), with the minimum and maximum ages of respondents being 18 and 53, respectively. The majority (67.5%) of food handlers were above 25 years of age. Of the total, 31% completed higher education. One hundred ninety-eight (49.5%) of the respondents had greater than 3 years of services in food establishments. More than half (53%) of respondents took formal training in food hygiene principles (Table 1).

### 3.2. Knowledge of Food Handlers on Food Safety

As shown in Table 2, all 400 participants (100%) knew that washing hand before handling food will reduce risk of poisoning, bacteria are the main cause of food poisoning, and temperature plays a big role in bacterial growth. Majority of the participants, 383 (95.8%), knew that raw food should be kept or stored separately from cooked food. Only 52.8% of the respondents knew that food can only be reheated once. In general, 65.5% (60.6%-70.2%) of food handlers had good knowledge about food safety, while 34.5% had poor food safety knowledge (Table 2). The mean score for knowledge of the respondents was 6.96 (SD = 1.053), and the minimum and maximum scores were five and eight, respectively, from the possible maximum score of eight.

### 3.3. Attitude of Food Handlers on Food Safety

The findings of the present study showed that 95.5% of participants believe that good personal hygiene can prevent foodborne illness. Additionally, 76% of the respondent are willing to attend training regarding food hygiene, 73.8% of the respondents believe they have a responsibility to practice safe food handling, and 52.8% of the respondents do not touch cooked foods (Table 3). The overall magnitude of good food safety attitude of food handlers was 65.3% (60.4%–69.9%) (Table 3). The mean score for the attitude of food handlers towards food safety was 14.93 (SD = 3.35), with minimum and maximum scores of 7 and 18, respectively, out of a possible maximum score of 18.

### 3.4. Practice of Food Handlers on Food Safety

As shown in Table 4, most of the participants had been practicing hand washing if got an abrasion, lesion, or cut; wash hands if scratching; and clean work station before and after start and finish works and 95.8% (383/400) of respondent had been to clean work station mop detergent, 92.3% (369/400) wash hands if after toilet visit, 91.8% wash hands if sneezing, and 83.5% (334/400) of participants have cover all the foods on the food stall. 83.8% (335/400) of the respondents thaw food by putting it in chill section in refrigerator, and 77.5% (310/400) of participants will take leave if continue coughing. Only 47% of the respondents will take leave if stomachache or cramps. The overall magnitude of good food safety practice of food handlers was 55.3% (50.2%-60.2%), and 44.7% of food handlers had poor food safety practice (Table 4). The mean score for practice of food handlers towards food safety was 20.38 (SD = 3.49), with minimum and maximum scores of 15 and 25, respectively, out of a possible maximum score of 25.

### 3.5. Association of Sociodemographic Characteristics with the Knowledge, Attitude, and Practice of Food Handlers

Even if food handlers with >25 years of age, higher education, 1-3 years of work experience, and permanent employment showed higher good knowledge about food safety, there was no statistically significant association between all sociodemographic factors and knowledge about food safety (Table 5).

As shown on Table 5, food handlers of >25 years of age and >3 years of experience have statistically significant good attitude towards food safety (*P* < 0.001). Food handlers who took formal training also have good attitude towards food safety (*P* = 0.014) (Table 5).

Food handlers with educational level of higher education have a statistically significant association for good food safety practice (*P* = 0.046). Food handlers who had >3 years of experience have statistically significant good practice towards food safety (*P* = 0.005). The status of food handlers showed a statistically significant association with food safety practice of food handlers (*P* = 0.004). Food handlers who took formal training also have a statistically significant good practice towards food safety (*P* = 0.01) (Table 5).

### 3.6. Association between Knowledge, Attitude, and Practice

As shown on Table 6, there was a statistically significant association between practice of food handlers with their knowledge and attitude towards food safety (*P* < 0.001).

### 3.7. Observational Assessment of Food Handlers on Food Safety

All (100%) food handlers wash their hands after using the toilet, and 90% keep uncooked foods separate from cooked food and take a medical checkup in the past six months. Majority (54.5%) of the respondents do not cover their hair while preparing food, and 45.5% of food handlers cover their hair while preparing food. Only 32.8% of food handlers wear any type of jewelry/ring on their hands at the time of the visit (Table 7). The overall good food safety practice of food handlers in the study area based on observational assessment was 38.5% (33.7%–43.5%) (Table 7). The mean score for practice of food handlers towards food safety was 14.12 (SD = 1.864), with minimum and maximum scores of 8 and 16, respectively, out of a possible maximum score of 17.

### 3.8. Association of Practice of the Respondents with Sociodemographic Factors Based on Observation

Based on observational assessment, the status of food handlers showed a statistically significant association with food safety practice of food handlers (*P* = 0.034) (Table 8).

## 4. Discussion

In the present study, 65.5% of food handlers had good knowledge about food safety. The present finding is higher than previous reports in Debre Markos town (34.1%) [24], Bole subcity of Addis Ababa (28%) [28], Dangila town (28.8%) [38], and Gondar town (44.3%) [39]. Similarly, this finding is higher than those reported in other countries such as India (58.3%) [40], Egypt (39.2%) [41], and Malaysia (51.6%) [42]. On the other hand, the present finding was slightly lower than the report in Southern Tigray (73.8%) [23], Bishoftu city (72.4%) [25], and Kenya (81.1%) [43].

In this study, the food handlers demonstrated knowledge regarding the significance of washing hands before handling food to reduce the risk of poisoning. They also acknowledged that bacteria are the primary culprits behind food poisoning and temperature plays a crucial role in bacterial growth. Comparable findings from studies conducted by [44] in Malaysia and [45] in Jordan indicated high percentage scores in respondents' knowledge concerning foodborne diseases, personal hygiene, and temperature regulations for food. The widespread understanding of these essential hygienic practices among the majority of institutional food handlers in this study holds significant importance. This is particularly crucial because the hands of food handlers can act as vectors in the transmission of foodborne diseases, either through poor personal hygiene or cross-contamination [46, 47]. Proper handwashing among food handlers has been documented to substantially reduce the risk of diarrheal diseases in childcare facilities [48], suggesting that encouraging such practices could similarly help mitigate the risks of diarrhea and other foodborne illnesses.

In the current study, 65.3% of the respondents have a good attitude towards food safety, which is in line with the findings of [23] (64.4%) in Southern Tigray [49] (64%) in Jigjiga town and [41] (61.2%) in Egypt. However, this result is higher than the reports of [28] (31%) in Bole subcity of Addis Ababa, [50] (54.8%) in Malesia, and [51] (33%) in Bangladesh. On the other hand, it is lower than the study of [25] (94.6%) in Bishoftu city and [45] (88.88%) in Jordan.

There is a statistically significant difference (*P* < 0.05) in attitude scores among some of the demographic profiles. Food handlers greater than 25 years old, more than 3 years of work experience, and taking formal training have a good attitude on food safety compared to their counterparts. Finding from our study is in opposite to the study of [52], which did not reveal difference in attitude scores among age, work experience, and training of food handlers.

In our study, the majority (95.3%) of food handlers believe that good personal hygiene can prevent foodborne illness (keep short nails, wash hands regularly, cover hair). Our result agrees with the result of [49] in which 93.4% food handlers believed that hand washing before handling meat reduces the risk of contamination. About 73.8% food handlers believed that practicing safe food handling is their responsibility. This result is supported by the finding of [53] in which 94.1% of the respondents agreed that preventing food contamination and spoilage is their key responsibility. Approximately 77.3% food handlers also believed that training on food safety could improve their food handling practice. Like our finding in a study by [49], 89.1% food handlers agreed that regular training could improve meat safety and hygiene practices. Similarly, [52] reported that about 94.7% food handlers agreed on the importance of food hygiene training to reduce risk of contamination. Our study showed that 73.3% of participants agreed that they should use different chopping boards for vegetables and meat. This result was much higher than the finding of [54] in which only 6.66% of the respondents agreed that raw vegetables and meat should not be cut in the same cutting board.

This study revealed that 55.3% has good food handling practice. This finding is comparable to the reports of [24] (54%) in Debre Markos town, [32] (55.1%) in Mettu and Bedelle towns, [55] (54.7%) in Nigeria, [38] (52.2%) in Dangla, and [56] (52.4%) in Dire Dawa. However, this result is lower than other studies conducted from different towns in Ethiopia such as Bishoftu city (83.7%) [25], Bahir Dar (67.6%) [57], and Mekelle (63.9%) [58]. The present result is also higher than the report in southern Tigray (42.3%) by [23] and Yeka subcity, Addis Ababa (40.2%), by [30]. The variations observed could be attributed to differences in the study design, cutoff points, and the study's respective year.

Likewise, this finding is lower than the findings in other studies conducted in Malaysia (59.30%) [35] and Jordan (89.43%) [45]. The differences could be attributed to variations in study settings. The Malaysian study took place on a university campus, while the Jordanian study was conducted in a hospital. These institutions are presumed to have ample resources and appropriate setups for food handling practices compared to the establishments in the present study. Additionally, the education levels of food handlers in Malaysia and Jordan might contribute to the observed variation. The percentage of food handlers with a secondary school education and above was 77% and 94% in the Malaysia and Jordan studies, respectively, whereas in the current study, it was only 59%. As educational levels increase, food handlers tend to exhibit enhanced knowledge and a more positive attitude towards proper food handling practices [27].

In contrast, this result was higher than the reports of [28] (27.4%) in Bole subcity of Addis Ababa, [36] (46.5%) in Woldia town, [7] (40.1%) in Debark town, [37] (32.6%) in Arba Minch town, and [59] (30.3%) in Gondar, Ethiopia. This could be attributed to variations in the study year and the criteria for cutoff points. The study carried out in Gondar town was approximately nine years ago. Over this period, the globalization of information increased, providing food handlers with improved access to knowledge, which could contribute to the development of better food handling practices [59]. Additionally, the criteria for determining food handling practices differed, as the Gondar study used ranges (80–100%, good; 60–79%, fair; and <60%, poor), while the present study assessed food handling practices in two levels based on the mean score. Obviously, the cutoff point variation entirely alters the results of the study. In Arba Minch town, interviewees were mostly having primary school and below (68.66%) as compared to this study in which 41% of respondents involved had primary and below education level. This is because food handlers with lower levels of education are likely to possess insufficient knowledge and a less favorable attitude, making them less inclined to adhere to fundamental principles of proper food handling [56].

The findings of this study indicate that food handlers with postsecondary school education exhibit notably better food handling practices. This observation aligns with similar results from studies conducted in Bahir Dar [57], Dire Dawa [56], Addis Ababa [27], Italy [60], Jordan [45], Ghana [61], and Nigeria [62]. The correlation between education level and food handling practices is attributed to the idea that a deeper knowledge base can positively influence the adherence of food handlers to standard procedures, ultimately contributing to the maintenance of food safety [27, 45, 63, 64].

Food handlers with greater than three 3 years of work experience had significantly higher good food handling practice. Similar finding was reported by [7] in Debark town, [36] in Woldia town, [57] in Bahir Dar, and [59] in Gondar. This might be because experience enables food handlers to gain enhanced knowledge and skills in food handling practices.

Food handlers who underwent formal training exhibited significantly better handling practices compared to those without such training. This observation is consistent with findings from previous studies [37, 45, 57, 59]. This association may be linked to the idea that training in food handling practices can enhance a food handler's knowledge about foodborne illnesses and related matters [65, 66]. Consequently, such training enables them to gain a deeper understanding, recognize their responsibilities, and improve their skills in food handling practices [57].

Thus, the implementation of proper food handling practices was notably higher among study participants who possessed good knowledge of food safety compared to their counterparts. Individuals with good knowledge are presumed to exhibit a positive attitude, which is a crucial factor influencing practical application. This result aligns with similar results from studies conducted in Mekelle [58] and Dangila [38].

There was a statistically significant association between practices of food handlers with their attitude towards food safety. Individuals with a positive attitude are presumed to possess a solid foundation of good knowledge, which serves as the basis for acquiring skills or putting knowledge into practice. This is also evidenced by other studies in Gondar [59], Dire Dawa [56], and Debark [7].

## 5. Conclusion and Recommendations

This study provided important information about the food safety knowledge, attitude, and practices of food handlers in Lemi Kura subcity, Addis Ababa. More than half of the food handlers had good knowledge, attitude, and practices towards food safety. Educational level, work experience, status, training, knowledge, and attitude were identified as factors affecting food safety practice. Therefore, food handlers should attend formal education and proper training about the basic principle of food safety in order to improve their knowledge, attitude, and practice towards food safety.

## Figures and Tables

**Table 1:** Sociodemographic characteristics of food handlers in food establishments of Lemi Kura subcity, Addis Ababa, Ethiopia.

Characteristics	Category	Frequency	Percent (%)
Sex	Female	194	48.5
	Male	206	51.5

Age	18-21 years	35	8.8
	22-25 years	95	23.7
	>25 years	270	67.5

Educational level	No formal education	83	20.8
	Primary school	81	20.3
	Secondary school	112	28.0
	Higher education	124	31.0

Position	Butcher	92	23.0
	Cook	77	19.3
	Manager	93	23.3
	Preparation	69	17.3
	Others	69	17.3

Experience	<1 year	31	7.8
	1-3 years	171	42.7
	>3 years	198	49.5

Employment status	Contract	31	7.8
	Permanent	310	77.5
	Temporary	59	14.8

Training	Formal training	212	53.0
	No training	188	47.0

**Table 2:** Knowledge of food handlers about food safety in food establishments of Lemi Kura subcity, Addis Ababa, Ethiopia.

Variables	Category	Frequency	Percent (%)
Washing hand before handling food will reduce the risk of poisoning	Yes	400	100.0
	No	0	0

Raw food should be kept or stored separately from cooked food	Yes	383	95.8
	No	17	4.3

Raw food should be kept on lower shelf and ready food should be stored on upper shelf	Yes	372	93.0
	No	28	7.0

Bacteria are the main cause of food poisoning	Yes	400	100.0
	No	0	0

Temperature plays a big role in bacterial growth	Yes	400	100.0
	No	0	0

Improper thawing or reheating of food will increase the risk of contamination	Yes	354	88.5
	No	46	11.5

Food can only be reheated once	Yes	211	52.8
	No	189	47.2

Defrosted food cannot be frozen again	Yes	263	65.8
	No	137	34.3

Level of food safety knowledge	Good	262	65.5
	poor	138	34.5

**Table 3:** Attitude of food handlers towards food safety in food establishments of Lemi Kura subcity, Addis Ababa, Ethiopia.

Variables	Category	Frequency	Percent (%)
It is my responsibility to practice safe food handling	Always	295	73.8
	Sometimes	105	26.3
	Never	0	0

I am willing to attend training regarding food hygiene	Always	304	76.0
	Sometimes	52	13.0
	Never	44	11.0

I believe good personal hygiene can prevent foodborne illness (keep short nails, wash hands regularly, cover hair)	Always	381	95.3
	Sometimes	19	4.8
	Never	0	0

I wash my hands every time before handling foods, after a toilet visit, sneeze, and getting cuts	Always	291	72.8
	Sometimes	65	16.3
	Never	44	11.0

I do not touch cooked foods	Always	211	52.8
	Sometimes	141	35.3
	Never	48	12.0

I use different chopping boards for vegetables and meat	Always	293	73.3
	Sometimes	107	26.8
	Never	0	0

I always make sure raw foods are in good condition before cook	Always	281	70.3
	Sometimes	100	25.0
	Never	19	4.8

If I am provided with safe food handling practices guideline, I will surely follow all of it even without supervision of my superior	Always	233	58.3
	Sometimes	123	30.8
	Never	44	11.0

If food training is given, I would practice a better food handling	Always	309	77.3
	Sometimes	66	16.5
	Never	25	6.3

Level of food safety attitude	Good	261	65.3
	Poor	139	34.7

**Table 4:** Food safety practice of food handlers in food establishments in Lemi Kura subcity, Addis Ababa.

Variables	Category	Frequency	Percent (%)
I wash my hands if I sneeze	Yes	367	91.8
	No	33	8.3

I wash my hands after toilet visit	Yes	369	92.3
	No	31	7.8

I wash my hands if I got an abrasion, lesion, or cut	Yes	400	100.0
	No	0	0

I wash my hands if I scratch	Yes	400	100.0
	No	0	0

I wash my hands if I am handling food	Yes	364	91.0
	No	36	9.0

I will take leave if I continue coughing	Yes	310	77.5
	No	90	22.5

I will take leave if I have a fever	Yes	297	74.3
	No	103	25.8

I will take leave if I have a stomachache or cramps	Yes	188	47.0
	No	212	53.0

I will take leave if I have a flu	Yes	245	61.3
	No	155	38.8

I use these to clean workstation tablecloth	Yes	329	82.3
	No	71	17.8

I use these to clean workstation water	Yes	361	90.3
	No	39	9.8

I use these to clean workstation warm water	Yes	343	85.8
	No	57	14.2

I use these to clean workstation disinfectant	Yes	278	69.5
	No	122	30.5

I use these to clean workstation mop detergent	Yes	383	95.8
	No	17	4.3

Leftover food management: thrown away	Yes	347	86.8
	No	53	13.3

Leftover food management: refrigerated and reheated	Yes	363	90.8
	No	37	9.3

Leftover food management: eaten at home	Yes	293	73.3
	No	107	26.8

I thaw food by letting it at room temperature to defrost itself in a covered container	Yes	309	77.3
	No	91	22.8

I thaw food by letting it at room temperature to defrost itself in an open container	Yes	324	81.0
	No	76	19.0

I thaw food by putting it in under running tap water	Yes	276	69.0
	No	124	31.0

I thaw food by putting it in chill section in refrigerator	Yes	335	83.8
	No	65	16.3

I do not refreeze defrosted food	Yes	299	74.8
	No	101	25.3

I cover all the foods on the food stall	Yes	334	83.5
	No	66	16.5

I clean my workstation before and after I start and finish my works	Yes	400	100.0
	No	0	0

It is not necessary to use thermometer to determine suitable meat temperature to cook	Yes	240	60.0
	No	160	40.0

Level of food safety practice	Good	221	55.3
	Poor	179	44.7

**Table 5:** Association of sociodemographic characteristics with food handlers' knowledge, attitude, and practice.

Sociodemographic factors		Food safety knowledge		*X* ^2^	*P* value	Food safety attitude		*X* ^2^	*P* value	Food safety practice		*X* ^2^	*P* value
		Good	Poor			Good	Poor			Good	Poor		
Sex	Female	122	72	1.139	0.286	123	71	0.567	0.451	102	92	1.088	0.297
	Male	140	66			138	68			119	87		

Age	18-21 years	21	14	1.188	0.552	17	18	21.806	0.000	15	20	5.721	0.057
	22-25 years	66	29			47	48			46	49		
	>25 years	175	95			197	73			160	110		

Educational level	No formal education	51	32	7.561	0.056	55	28	6.989	0.072	39	44	7.998	0.046
	Primary school	54	27			62	19			52	29		
	Secondary school	65	47			71	41			55	57		
	Higher education	92	32			73	51			75	49		

Position	Butcher	66	26	8.229	0.084	56	36	1.639	0.802	55	37	5.912	0.206
	Cook	54	23			49	28			37	40		
	Manager	55	38			64	29			48	45		
	Preparation	38	31			45	24			36	33		
	Others	49	20			47	22			45	24		

Experience	<1 year	20	11	3.795	0.150	11	20	36.086	0.000	9	22	10.555	0.005
	1-3 years	121	50			94	77			93	78		
	>3 years	121	77			156	42			119	79		

Employment status	Contract	26	5	5.686	0.058	22	9	2.065	0.356	23	8	10.995	0.004
	Permanent	201	109			205	105			175	135		
	Temporary	35	24			34	25			23	36		

Training	No training	125	63	0.154	0.695	111	77	6.028	0.014	91	97	6.724	0.010
	Formal training	137	75			150	62			130	82		

**Table 6:** Association between knowledge, attitude, and practice.

Factors		Practice level		*X* ^2^	*P* value
		Good	Poor		
Attitude level	Good	177	84	47.969	0.000
	Poor	44	95		

Knowledge		194	68	108.512	0.000
		27	11		

**Table 7:** Observational assessment on food handlers towards food safety.

Variables	Category	Frequency	Percent (%)
Do food handlers wear outer garments or gowns during the visit	Yes	182	45.5
	No	218	54.5

If they wear outer garments or gowns, do the garments or gowns were clean	Yes	182	45.5
	No	218	54.5

Do food handlers cover their hair while working	Yes	182	45.5
	No	218	54.5

Do food handlers' fingernails short trimmed and clean	Yes	360	90.0
	No	40	10.0

Do food handlers wear any type of jewelry/ring on their hands at the time of the visit	Yes	131	32.8
	No	269	67.3

Clean the work surfaces after each task	Yes	372	93.0
	No	28	7.0

Used soap/detergent for washing dishes	Yes	400	100
	No	0	0.0

Used hot water for washing dishes	Yes	360	90.0
	No	40	10.0

Wash their utensils using three washing compartments	Yes	400	100.0
	No	0	0.0

Did food handlers wash the chopping board and knife with soap or bleach after using	Yes	359	89.8
	No	41	10.3

Did food handlers wash their hands with detergent and water before working with food	Yes	400	100.0
	No	0	0.0

Did food handlers wash their hands with detergent and water after visiting the toilet	Yes	400	100.0
	No	0	0.0

Did food handlers keep ready-to-eat foods in a hygienic container	Yes	359	89.8
	No	41	10.3

Did food handlers carefully keep food utensils on the shelf/cabinet	Yes	399	99.8
	No	1	0.3

Did food handlers keep uncooked foods separate from cooked food	Yes	360	90.0
	No	40	10.0

Did food handlers take a medical checkup in the past six months	Yes	360	90.0
	No	40	10.0

Does the establishment and food handlers were inspected by regulatory personnel in the past six months	Yes	360	90.0
	No	40	10.0

Level of observed food safety practice	Good	154	38.5
	Poor	246	61.5

**Table 8:** Association between practice of food handlers towards food safety and sociodemographic characteristics of the respondents based on observation.

Sociodemographic factors		Food safety practice based on observation		*X* ^2^	*P* value
		Good	Poor		
Sex	Female	70	124	0.930	0.335
	Male	84	122		

Age	18-21 years	10	25	1.6131	0.446
	22-25 years	38	57		
	>25 years	106	164		

Educational level	No formal education	27	56	1.784	0.619
	Primary school	33	48		
	Secondary school	43	69		
	Higher education	51	73		

Position	Butcher	33	59	2.012	0.734
	Cook	27	50		
	Manager	35	58		
	Preparation	31	38		
	Others	28	41		

Experience	<1 year	9	22	3.655	0.161
	1-3 years	60	111		
	>3 years	85	113		

Employment status	Contract	17	14	6.760	0.034
	Permanent	121	189		
	Temporary	16	43		

Training	No training	68	120	0.813	0.367
	Formal training	86	126		

## Data Availability

All relevant data are contained within the article.
